# Supplementary material for: Chiral acidic amino acids induce chiral hierarchical structure in calcium carbonate
Source: Nat Commun. 2017 Apr 13;8:15066. doi: 10.1038/ncomms15066 (PMC5399303; doi:10.1038/ncomms15066)
Supplement: Supplementary Information — Supplementary Figures, Supplementary Table, Supplementary Discussion and Supplementary References. [file ncomms15066-s1.pdf]

Supplementary Discussion

Docking on alternate vaterite models and surfaces

Mechanism "A": Chiral amino acid adsorption on symmetric vaterite. We sought to construct a model of L- and D-Asp and Glu amino acid binding to a vaterite surface that would explain the emergence of chiral nanoparticle-based suprastructures, taking into account the dominant lattice *d*-spacing (0.36 nm) obtained from X-ray diffraction (XRD) and high-resolution transmission electron microscopy (TEM) (Supplementary Figs. 1I and 9). We examined several hypotheses for vaterite structures (Supplementary Table 1). We first selected the recent 2009 crystal structure of vaterite by Wang and Becker[1] based on quantum chemical calculations. The (110) surface of this structure matches the experimental *d*-spacing we observed. Initial docking runs identified regions of local symmetry on the (110) surface where chiral amino acids adsorbed in mirror-image orientations. However, the binding-energy calculations differed considerably, although the enantiomeric structures were similar on the solvent-exposed surface; asymmetry outside of the first shell of contacts caused significant differences in binding energies. This initial result was inconsistent with the experimental data, where both L- and D-Asp affect the crystal habit identically but with opposite chirality.

With this inconsistency in mind, we refined our model to require a mirror or glide plane on the vaterite surface so that iso-energetic binding of chiral amino acids would be possible. In our refined model, chiral amino acids adsorb to an initially achiral surface and produce a chiral organic monolayer that is capable of directing subsequent crystal growth in an ordered fashion to produce chiral suprastructures. In order for the L- and D-enantiomers to create symmetric chiral suprastructures, the mineral-binding plane must have a symmetry element such as a mirror or glide plane. Thus, for the next set of models, we explored the 1963 crystal structure presented by Kamhi[2], which is based on powder diffraction patterns and has $P6_3/mmc$ symmetry. The (100) surface of Kamhi's structure matches the experimental *d*-spacing we observed, and it has a mirror plane passing through it orthogonally, meaning that chiral amino acid adsorption will necessarily break the symmetry. A challenge in Kamhi's structure is that the carbonate groups have partial occupancy, indicating disorder in their arrangement. In this study we considered "vertical" and "angled" orientations of the two carbonate groups in the unit cell. The "vertical" carbonate orientations give rise to a second mirror plane orthogonal to the first. The second mirror plane removes any oriented preference a chiral amino acid may have because for every binding site on the surface, an identical binding site exists when the structure is rotated by 180°. Thus,

the modified Kamhi model with vertical carbonates is also inconsistent with our study's chirality observations.

With one orthogonal mirror plane, the (100) surface with "angled" carbonate groups presents the necessary symmetry. There are several possibilities for the termination of the (100) surface. Since the amino acids are negatively charged, and local electrostatic complementarity is needed for favorable binding energies, we discarded the possibility of a net-negatively charged surface termination. We next explored a net-positively charged surface termination and found that it both maintained surface symmetry and strongly adsorbed negatively charged acidic amino acids. However, because of the even spacing of calcium ions on the net-positively charged surface, rotation of any acidic amino acid by 180° creates similar electrostatic contacts between both carboxyl groups and surface calcium atoms. Because these energetically favorable contacts can be made regardless of amino acid orientation, adsorption on net-positively charged surfaces cannot explain the different chiral morphologies resulting from chiral amino acid adsorption.

Finally, we investigated net-neutrally terminated surfaces because they often have low surface energies and are stable in solution (Supplementary Fig. 10). We explored several net-neutrally terminated surfaces by deleting alternating Ca atoms as well as alternating rows of Ca atoms. We found that deleting alternating rows of Ca atoms perpendicular to the mirror plane maintained symmetry and provided an environment where directionally dependent interactions could be made, yielding an orientational preference. Given that the net-neutrally terminated modified Kamhi (100) surface is expected to be stable in solution, maintains symmetry, and has the potential for orientational preference, we anticipated that docking simulations performed on this surface might generate models capable of explaining the different chiral morphologies resulting from chiral amino acid adsorption. Indeed, as described in the main text, on the modified Kamhi (100) surface, L-Asp and D-Asp have preferred orientations of adsorption, and adsorption in the orientation of the opposite enantiomer changes the binding energy by 0.64 kcal mol^{-1}. In an ensemble of molecules, this energy difference would result in one orientation being approximately three times as likely as the other, which could create the 4° tilt between nanoparticles observed in the vaterite suprastructure.

In addition to considering how the bulk crystal should be terminated, we also considered the possibility of hydrated species existing at the vaterite surface as a function of pH, as has been found to be important in other systems[3]. Our experiments confirm the formation of chiral morphology from pH 6.46 to 12.40 (Fig. 3 in main text). In solution, across the pH range 6.46 to 12.40, carbonate is present in

both the HCO_3^- and CO_3^{2-} forms based simply on the pK_a of carbonic acid[4]. Unfortunately, there are no experiments that probe the hydration species present on any vaterite surface. On a calcite [104] surface, AFM studies from Stipp[5] suggest the presence of stable hydration species $CaOH^+$ and HCO_3^-. However, AFM studies on calcite from Ohnesorge & Binnig[6] and Rachlin *et al.*[7] suggest that a pristine bulk termination exists. Another AFM study from Liang *et al.* [8] observed a pH-independent AFM image between pH range 4-9, suggesting the absence of pH-dependent hydration species. The invariance of our observations across a wide range of pH suggests two possibilities. First, a pristine termination may persist for the entire pH range. This is the case represented by our structure prediction models. As shown in Fig. 6a of the main text, a strong hydrogen bond between the amino terminus of Asp and a surface carbonate ion is needed for chiral selectivity. In the second case, at lower pH, the hydrated HCO_3^- ion may be important. A pH-dependent change in the concentration of hydrated species and restructuring of the surface could be complemented by a change in the protonation state of the amino acid. The same hydrogen bonding geometry can be achieved whether the proton is attached to the surface carbonate or to the amino terminus of Asp. This alternate chemistry was used to confirm the plausibility of this structure by simple minimization calculations.

In summary, our model “A” for docking simulations explains the experimentally observed symmetry breaking in terms of chiral amino acids adsorbing onto an achiral surface. Under these conditions a valid model must meet the following criteria: (1) match the TEM and XRD *d*-spacings, (2) provide electrostatic complementarity to a negatively charged acidic amino acid, (3) contain a single mirror or glide symmetry plane, and (4) provide a chemically diverse binding patch to provide oriented preference. Out of the possible vaterite models we explored, only the “angled” positively terminated (100) surface of Kamhi (1963) matched all criteria.

Mechanism “B”: Chiral amino acid adsorption on chiral vaterite. A second potential explanation for the observed chiral vaterite suprastructures is that chiral amino acids may preferentially nucleate one chiral crystal enantiomer over the other. Thus, as an alternative strategy, we investigated a second potential mechanism based on a recent vaterite structural model by Demichelis *et al*[9]. Demichelis *et al.* propose several pairs of chiral structures for vaterite. We chose to use Demichelis *et al.*’s lowest energy pair of chiral vaterite structures, which belong to the space groups $P3_221$ and $P3_121$. The (110) surface of these structures matches our experimentally observed *d*-spacings. Similarly to the modified Kamhi (100) surface, we considered negative, positive, and neutral terminations of the

Demichelis *et al*. (110) surfaces. As with the modified Kamhi (100) surface, we selected a neutral termination of the Demichelis $P3_121$ (110) surface for docking studies (Fig. 9b in main text). Docking of both L- and D-Asp onto the neutrally terminated Demichelis $P3_121$ (110) surface revealed that binding of L-Asp onto the $P3_121$ surface is favored by 0.33 kcal mol^{-1} over D-Asp (which, by symmetry, implies binding of D-Asp onto the $P3_221$ is favored by 0.33 kcal mol^{-1} over L-Asp). The difference in adsorption energy arises from the electrostatic complementarity between the $P3_121$ surface and L- and D-Asp (Fig. 9 in main text). Both L- and D-Asp are anchored to the surface via hydrogen bonding between the amino terminus and the surface carbonates as well as by electrostatic contacts between the carboxyl terminus and one surface calcium. With these two functional groups locked in identical positions for both L- and D-Asp, the adsorption energy difference is attributable to the interaction of the charged side chain with the mineral. In the case of L-Asp, the side chain carboxyl group is able to interact favorably with an exposed calcium ion. However, in the case of D-Asp, the side chain carboxyl is forced to interact unfavorably with a highly coordinated calcium ion.

Comparison of proposed mechanisms

Both our "A" and "B" models provide a potential mechanism for the experimentally observed chiral morphologies resulting from vaterite growth in the presence of chiral amino acids. Many potential structural models of vaterite have been proposed in the literature[1,2, 9,10-13], and our amino acid adsorption models are limited by the conception of the vaterite atomic structure. Our primary model "A" relies on symmetry breaking at the mineral surface. Differing interfacial structures with adsorbed chiral amino acids stabilize one nanoparticle tilt direction over the other. In our secondary model "B", symmetry breaking occurs by selecting a chiral form of the bulk crystal. Both models match the experimental *d*-spacings that we observed. A potential problem with the primary model is that the stability of the 1963 Kamhi vaterite structure has been controversial in the recent literature[9,10,13] and does not provide specific coordinates for the atoms of the carbonate group. An advantage of the primary model is that the difference in adsorption energies at alternate sites is 0.64 kcal mol^{-1}, which is nearly double that of the secondary model.

Supplementary Figures

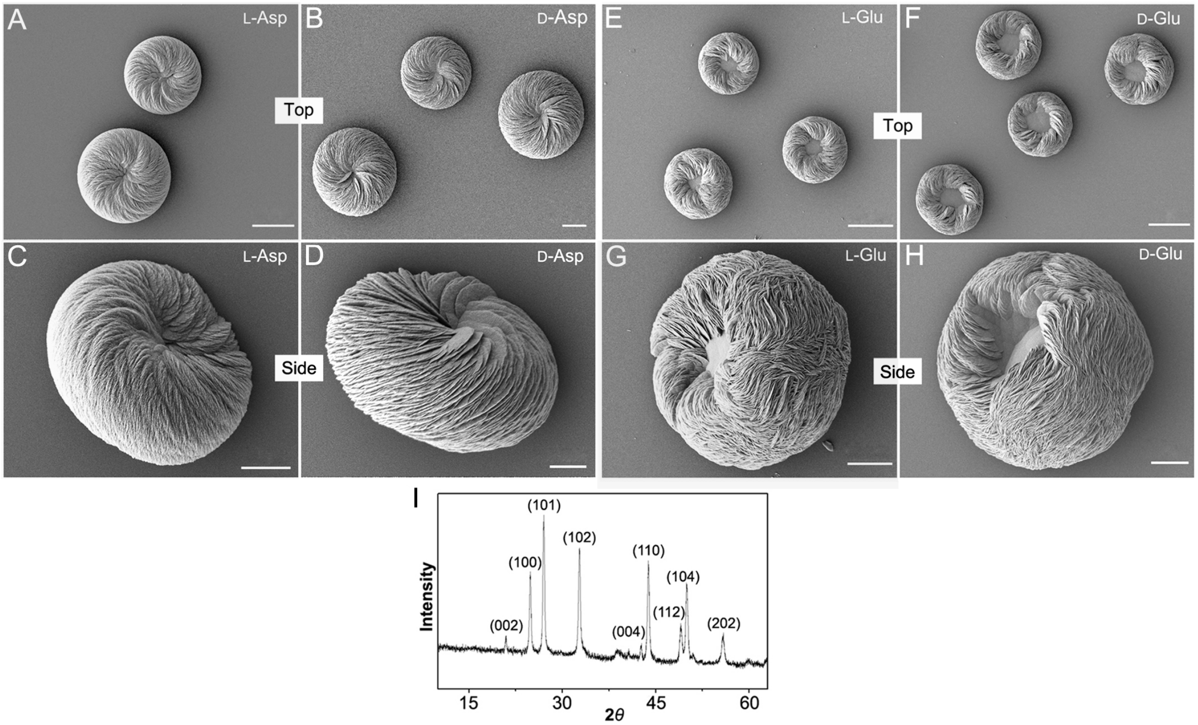

Supplementary Figure 1 │ Chiral hierarchical vaterite toroids induced by chiral acidic amino acids. SEM images showing top and side views of toroid morphology after growth in the presence of 20 mM Asp and Glu enantiomers for 24 h where L-Asp (**A**, **C**) and L-Glu (**E**, **G**) create a counterclockwise (right-handed) orientation of the suprastructure, and D-Asp (**B**, **D**) and D-Glu (**F**, **H**) create a clockwise (left-handed) orientation of the suprastructure. (**I**) X-ray diffraction pattern characteristic for vaterite, here shown for supersaturated solution growth in the presence of L-Glu. Similar results were obtained for calcium carbonate growth in the presence of all chiral enantiomers for Asp and Glu. During growth, central cores consist of the initial discs that at the outset consist of amorphous calcium carbonate (see Figure 6 in Main Text, Supplementary Figure 2, and Supplementary Movies 1 and 2). Scale bars: 10 µm (**A**, **B**), 5 µm (**C**, **D**), 40 µm (**E**, **F**), and 15 µm (**G**, **H**).

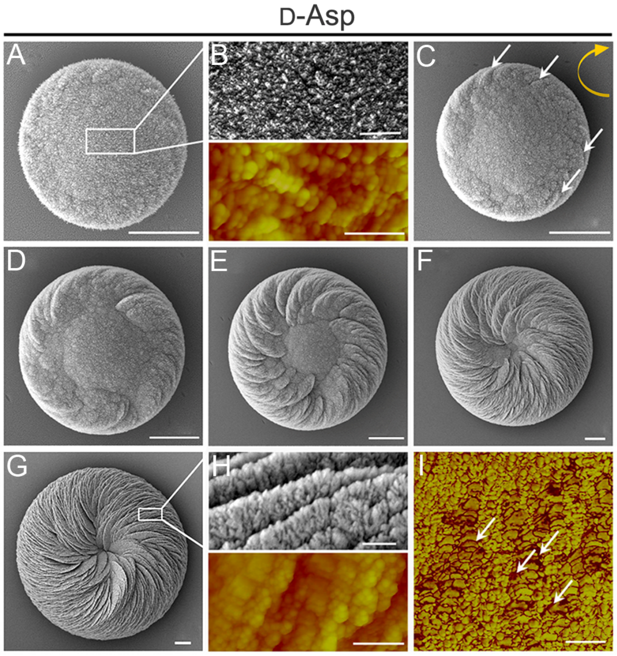

Supplementary Figure 2│ Growth evolution of chiral vaterite toroids grown in D-Asp or D-Glu. SEM and AFM (colored) images of vaterite toroid growth for 2 h (**A**, **B**), 3 h (**C**), 5 h (**D**), 8 h (**E**), 12 h (**F**) and 24 h (**G**, **H**) in the presence of D-Asp, and for 1.5 h (**J**, **K**), 2 h (**L**), 3 h (**M**), 5 h (**N**), 10 h (**O**) and 24 h (**P**, **Q**) in the presence of D-Glu. Oriented vaterite platelets (white arrows) emerge within several hours (**C**, **L**) at the outer edge of a flat substrate vaterite disc to begin the formation of a toroid with a counterclockwise chirality (green curved arrow), and these and other platelets continue spiral growth to encroach upon, and then obscure (in the case of Asp), the centrally located achiral vaterite core region. AFM height images (**B** and **H**, and **K** and **Q**, lower panels) show that all vaterite elements in the toroids have nanoparticle substructure, which is aligned in the platelets, and which in the AFM phase mode (**I**, **R**) shows inorganic vaterite nanoparticles (yellow) surrounded by organic amino acid (red). Scale bars: 3 µm (**A**, **C**-**G**), 400 nm (upper panel of **B**), 300 nm (lower panel of **B**), 500 nm (upper panel of **H**), 400 nm (lower panel of **H**), 300 nm (**I**), 10 µm (**J**, **L**-**P**), 400 nm (upper panel of **K**), 200 nm (lower panel of **K**), 1 µm (upper panel of **Q**), 200 nm (lower panel of **Q**), and 300 nm (**R**).

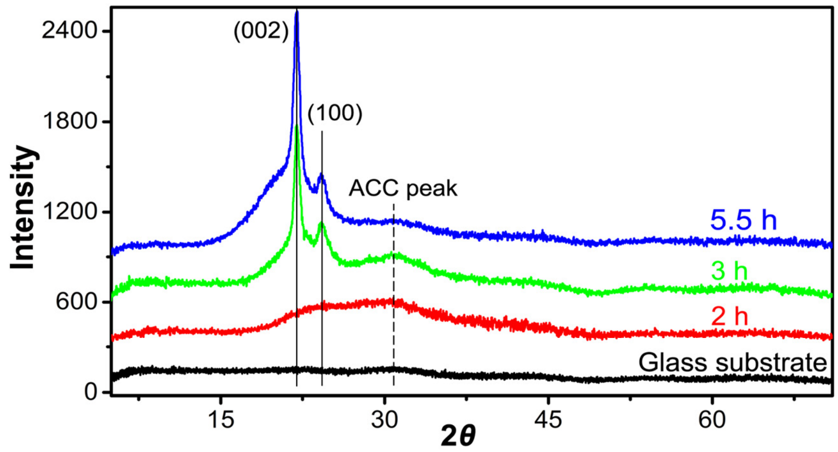

Supplementary Figure 3 | Mineral phase evolution of chiral toroids grown in L-Asp. X-ray diffraction (XRD) pattern characteristic of early-stage calcium carbonate immature initial discs (without platelets) grown for 2 hours on a glass coverslip substrate show only a single very broad peak at about 31° of 2 theta (vertical dashed line), indicative of ACC, as also observed by Micro-Raman spectroscopy and SEM (Main Text Figs. 5 and 6). With further toroid growth, two sharp crystallized vaterite peaks appeared, the (002) and (100) planes (two vertical solid lines), whose intensity increased with time, while the broad ACC peak decreased. These XRD data indicate a phase transformation from the amorphous state to crystalline vaterite, which is exactly what was observed by Micro-Raman spectroscopy (Main Text Fig. 6).

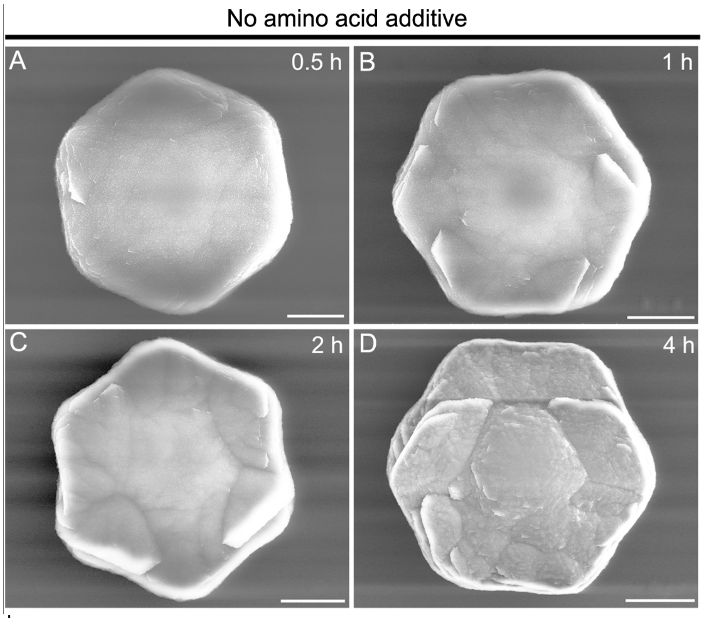

Supplementary Figure 4 │ Symmetric (achiral) vaterite formed over time in the absence of amino acids. SEM images showing that characteristic hexagonal vaterite forms in supersaturated growth solution in the absence of amino acid after 0.5 h (**A**), 1 h (**B**), 2 h (**C**) and 4 h (**D**). Scale bars: 4 μm (**A**), 6 μm (**B**), 8 μm (**C**), and 12 μm (**D**).

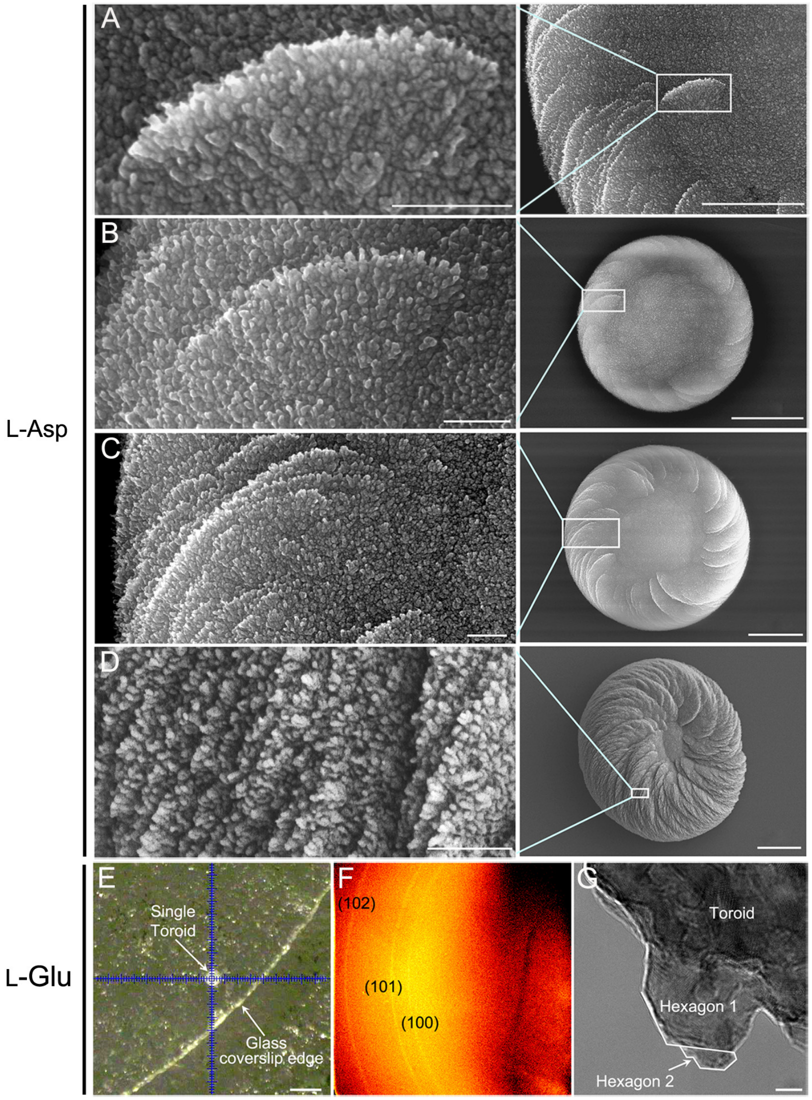

Supplementary Figure 5 │ Polycrystalline nanostructure of vaterite toroids. (**A-C**) High-magnification SEM of the rounded platelet edges of a chiral toroid formed in the presence of L-Asp showing layered platelet growth and curved platelet edges, all occurring via a nanostructured growth mechanism. (**D**) On-edge view of platelets showing that a single layer of aligned nanoparticles forms the platelet thickness of a toroid formed in the presence of L-Asp. (**E**) Optical image of single toroid (in cross-hairs) grown in the presence of L-Glu as analyzed by (**F**) X-ray diffraction (XRD) showing diffraction maxima (rings) indicative of a polycrystalline material in L-Glu rather than a spot pattern that would be obtained from a single crystal. (**G**) TEM image from the edge of a toroid platelet grown in L-Glu showing a "daughter" nanohexagon (Hexagon 2) immediately adjacent to its "mother" nanohexagon (Hexagon 1) at the edge of a platelet, indicating nanoparticle growth occurs upon existing nanoparticles. Scale bars: 400 nm (left panels of **A-D**), 2 μm (right panels of **A**), 4 μm (right panels of **B-D**), 200 μm (**E**), and 7 nm (**G**).

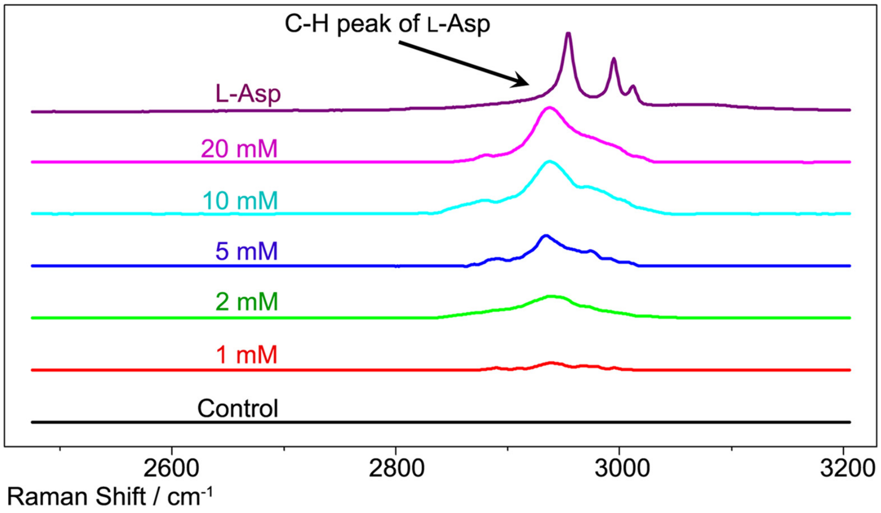

Supplementary Figure 6 │ Incorporation of chiral acidic amino acids into chiral vaterite toroids. Micro-Raman spectra of pure L-Asp and vaterite toroids formed in the presence of L-Asp at different concentrations in the supersaturated calcium carbonate solution, which are normalized to the peak of ν_1 symmetric stretching of carbonate groups at 1089 cm^{-1}. The C-H peak of L-Asp seen by micro-Raman spectroscopy of the chiral toroids deviated by about 20 cm^{-1} compared to that of pure L-Asp solid powder, suggesting that chiral acidic amino acids were extensively incorporated into the platelets. These peaks increase with increasing concentration of L-Asp. Similar results were obtained for all enantiomers of Asp and Glu.

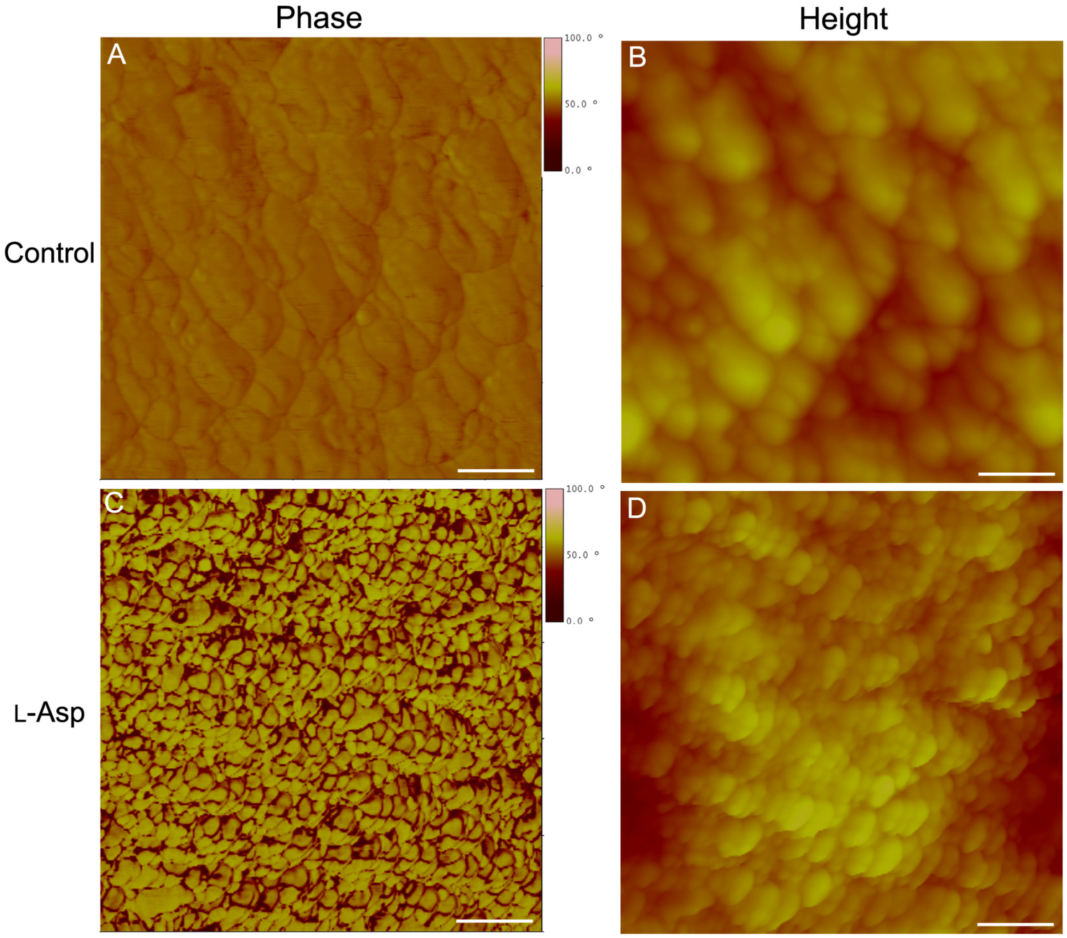

Supplementary Figure 7 │ Organic-inorganic hybrid structure of vaterite platelets in toroids. While both chiral toroids (with amino acid, L-Asp) and achiral hexagonal vaterite crystals (without amino acid, Control) have similar nanostructure by AFM height imaging (**B**, **D**), AFM phase imaging (**A**, **C**) clearly shows vaterite nanoparticles (yellow). In the absence of amino acid, AFM height (**B**) and phase (**A**) images show the similar nanostructured morphologies of achiral hexagonal vaterite crystal. However, in the presence of L-Asp, the AFM height (**D**) image shows the nanostructured morphology of the vaterite platelets of the toroids, whereas the AFM phase image of a platelet (**C**) obviously displays two different compositions and is very different from the height image, and indicates changes in composition rather than the morphology of platelets. Scale bars: 200 nm (**A-D**).

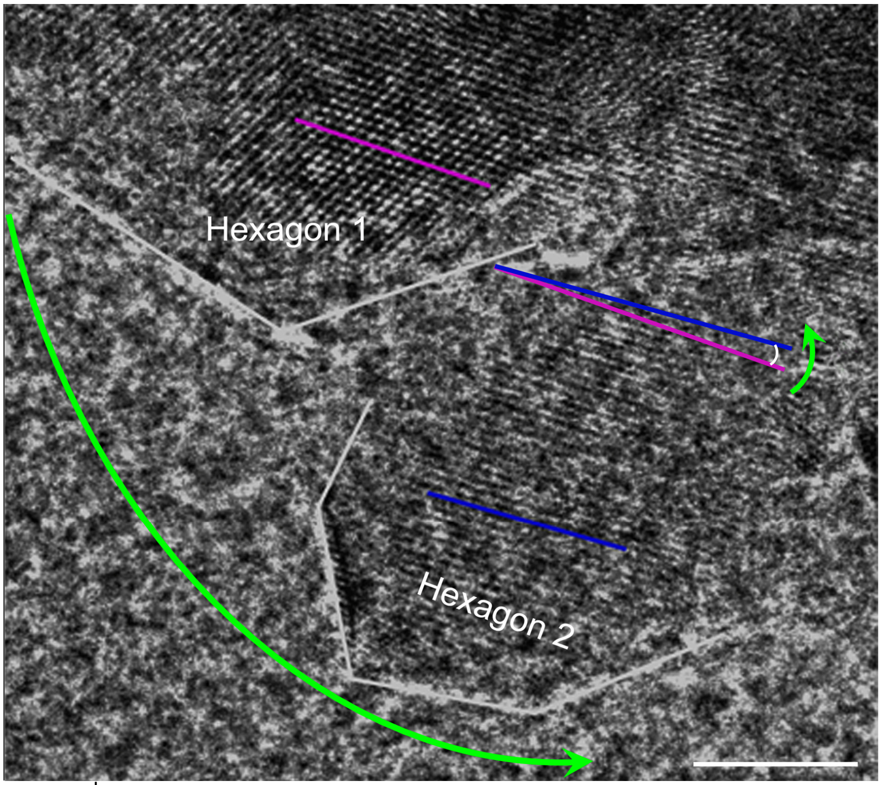

Supplementary Figure 8 │ Configuration of two consequential nanohexagons in a vaterite platelet. High-resolution TEM image showing two adjacent hexagons with their internal lattice structure in a vaterite platelet having a counterclockwise direction (long green arrow) formed in the presence of L-Asp. For the consequential "daughter" hexagon (Hexagon 2), its (100) plane (blue line) is tilted by approximately -3º (in the counterclockwise direction, short green arrow) relative to the same plane (purple line) in the "mother" hexagon (Hexagon 1). Scale bar: 6 nm.

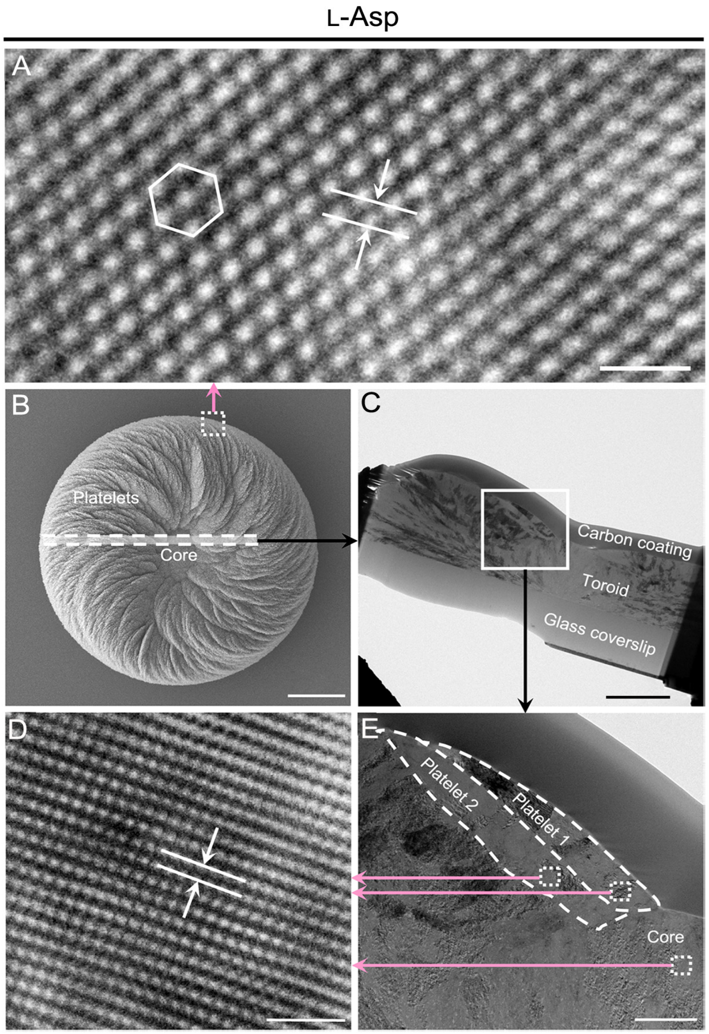

Supplementary Figure 9 │ Atomic structure of vaterite platelets in chiral toroids. (**A**) High-resolution TEM image showing vaterite platelets formed in the presence of L-Asp. On the basis of hexagonal symmetry considerations (*P6₃/mmc*) of the lattice points, the (001) face should be the exposed facet plane, and all the *d*-spacings of the hexagonal lattice are 0.36 nm (arows), which confirms that platelets originate and propagate along the (100) plane. (**B**-**E**) Inner structure of a vaterite toroid after focused-ion beam (FIB) cutting. (**B**)SEM image showing an intermediate-stage toroid formed in the presence of L-Asp before FIB cutting; the dashed rectangular area demarcates the region from which the FIB slice was taken. **C**, Low-magnification TEM image showing the cross-sectional FIB slice of the toroid composed of the glass coverslip substrate on which the toroid grew, the toroid itself, and the protecting carbon layer coating deposited as part of the FIB cutting process. (**D**, **E**) Higher magnification TEM images showing the cut toroid with vaterite lattice structure in both the central core region and in the platelets of (001) planes (*d* = 0.42 nm, arrows), this being consistent with the hexagonal symmetry in panel (A). Scale bars: 1 nm (**A**), 4 μm (**B**, **C**), 2 nm (**D**) and 1 μm (**E**).

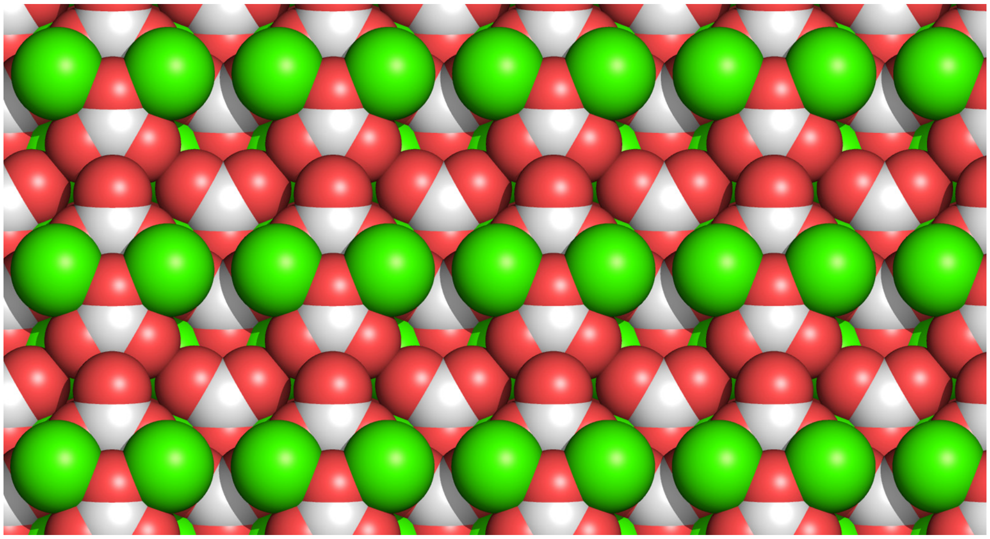

Supplementary Figure 10 | Symmetric vaterite surface. Atomic structure of the net-neutrally terminated, symmetrical modified Kamhi (100) plane (primary model "A"). Ca, green; C, grey; O, red.

Supplementary Table

Supplementary Table 1: Vaterite surfaces

Unit Cell Coordinates	Miller Indices	CO_3 Orientation	Termination	Surface Symmetry	Notes
Wang and Becker (2009) *P6₃/522*	110	N/A	neutral	local mirror but not global	no global mirror plane
Kamhi (1963) *P6₃/mmc*	110	N/A	N/A	no mirror	*d*-spacing does not agree with our experimental observations
	100	vertical	positive	mirror/mirror	2nd mirror plane negates oriented preference
			neutral (rows)	mirror/mirror	2nd mirror plane negates oriented preference
			neutral (checkerboard)	mirror/mirror	2nd mirror plane negates oriented preference
			negative	mirror/mirror	negative surface unlikely to bind negatively charged amino acids
		angled	positive	mirror	L- and D-enantiomers show no orientation preference
			neutral	mirror	L- and D-enantiomers break symmetry (Model "A")
			negative	mirror	negative surface unlikely to bind negatively charged amino acids
Demichelis *et al. (2013) P3₁21*	110	N/A	neutral	no mirror (chiral surface)	L-Asp adsorption is favored (Model "B")
Demichelis *et al. (2013) P3₂21*	110	N/A	neutral	no mirror (chiral surface)	D-Asp adsorption is favored (Model "B")

Supplementary References

1. Wang, J. W. & Becker, U. Structure and carbonate orientation of vaterite ($CaCO_3$). *Am. Mineral.* **94,** 380-386 (2009).
2. Kamhi, S. On the structure of vaterite $CaCO_3$. *Acta Crystallogr.* **16,** 770-772 (1963).
3. Heinz, Z. The role of chemistry and pH of solid surfaces for specific adsorption of biomolecules in solution—accurate computational models and experiment. J. Phys.: Condens. Matter **26,** 244105 (2014).
4. Nakayama, F. S. Sodium bicarbonate and carbonate ion pairs and their relation to the estimation of the first and second dissociation constants of carbonic acid. *J. Phys. Chem.* **74,** 2726–2728 (1970).
5. Stipp, S. L. S. Toward a conceptual model of the calcite surface: hydration, hydrolysis, and surface potential. *Geochimica et Cosmochimica Acta* **63,** 3121–3131 (1999).
6. Ohnesorge, F. & Binnig, G. True atomic resolution by atomic force microscopy through repulsive and attractive forces. *Science* **260,** 1451-1456 (1993).
7. Rachlin, A. L., Henderson, G. S. & Goh, M. C. An atomic force microscope (AFM) study of the calcite cleavage plane; image averaging in fourier space. *Am. Mineral.* **77,** 904–910 (1992).
8. Liang, Y., Lea, A. S., Baer, D. R. & Engelhard, M. H. Structure of the cleaved $CaCO_3$ (1014) surface in an aqueous environment. *Surf Sci.* **351,** 172–182 (1996).
9. Demichelis, R., Raiteri, P., Gale, J.D. & Dovesi, R. The multiple structures of vaterite. *Cryst. Growth Des.* **13,** 2247–2251 (2013).
10. Demichelis, R., Raiteri, P., Gale, J. D. & Dovesi, R. A new structural model for disorder in vaterite from first-principles calculations. *Crystengcomm* **14,** 44-47 (2012).
11. De La Pierre, M., *et al.* Probing the multiple structures of vaterite through combined computational and experimental Raman spectroscopy. *J. Phys. Chem. C* **118,** 27493-27501 (2014).
12. Mugnaioli, E., *et al.* Ab initio structure determination of vaterite by automated electron diffraction. *Angew. Chem. Int. Ed.* **51,** 7041-7045 (2012).
13. Behrens, G., Kuhn, L. T., Ubic, R. & Heuer, A. H. Raman-spectra of vateritic calcium-carbonate. *Spectrosc Lett.* **28,** 983-995 (1995).
